# Dietary patterns associated with benign breast nodules by subtypes: a cross-sectional study in southeast China

**DOI:** 10.3389/fnut.2025.1500853

**Published:** 2025-03-25

**Authors:** Pingxiu Zhu, Mei He, Jiamin Gong, Qingling Su, Ruimei Feng, Yundan Cai, Weihong Qiu, Haomin Yang, Shanshan Du, Weimin Ye

**Affiliations:** ^1^Department of Epidemiology and Health Statistics, School of Public Health and Key Laboratory of Ministry of Education for Gastrointestinal Cancer, Fujian Medical University, Fuzhou, China; ^2^Department of Ultrasonography, Fuqing City Hospital Affiliated to Fujian Medical University, Fuqing, China; ^3^Institute of Population Medicine, School of Public Health, Fujian Medical University, Fuzhou, China; ^4^Department of Epidemiology, School of Public Health, Shanxi Medical University, Taiyuan, China; ^5^Department of Medical Epidemiology and Biostatistics, Karolinska Institutet, Stockholm, Sweden

**Keywords:** dietary patterns, breast benign nodules, odds ratio, cross-sectional study, animal-based dietary pattern, fried food/dessert pattern

## Abstract

**Background:**

Dietary patterns influence women's risk of breast cancer, but few studies have investigated the association with benign breast nodules, a well-established risk factor for breast cancer, especially by subtypes of the disease.

**Methods:**

A cross-sectional study of 3,483 women enrolled in the Fuqing Cohort Study in southeast China was conducted from 2020 to 2021. Dietary patterns were identified from food frequency questionnaires using principal component analysis, and the scores for these patterns were divided into quartiles. Univariate and multivariate logistic regression models were used to calculate odds ratios (OR) with 95% confidence interval (CI) for the association between dietary patterns and benign breast nodules.

**Results:**

We found four dietary patterns among the women: animal-based dietary pattern, plant-based dietary pattern, fried food/dessert pattern, and nuts pattern. Compared with the lowest quartile, women in the highest quartile of the scores for the animal-based dietary pattern were more likely to have cystic breast nodules (OR = 1.61, 95% CI = 1.12–2.32, and the *P*-value for trend test = 0.007), especially in postmenopausal women. In addition, women with a high score for fried food/dessert pattern also had higher odds of breast cystic nodules (*P*-value for trend test = 0.012), with an OR (95% CI) of 1.46 (1.01–2.09) for the fourth quartile group. However, there were no associations between these dietary patterns and solid breast nodules.

**Conclusion:**

Animal-based dietary pattern and fried food/dessert pattern were positively associated with cystic breast nodules. These findings suggested the role of unhealthy dietary habits in the development of breast nodules.

## Introduction

Benign breast nodules are the most common breast disease in women (1). Although benign breast nodules are not lethal diseases themselves, they accounts for 75% of breast biopsy diagnoses (2). Notably, patients with benign breast nodules have a significantly higher risk of breast cancer than women without breast nodules (3), and breast cancer is a leading cause of cancer mortality among women.

The etiology of benign breast nodules involves a complex interaction of genetic and environmental risk factors (4). Risk factors for benign breast nodules are similar to the known risk factors for breast cancer (5), including dietary habits, lack of physical activity, age at menarche, number of births, and family history of breast cancer (6).

As one of the few modifiable risk factors for breast diseases, previous epidemiological studies suggest a significant contribution of dietary patterns to the risk of benign breast nodules (7–9). However, these studies are limited by small sample sizes, or by focusing on a selected subgroup of women, which might affect the variety of dietary patterns identified. Furthermore, to date, no study has examined the influence of dietary patterns on various subtypes of benign breast nodules and according to menopausal status, which may vary in their pathological processes.

With the improvement of living standards in recent years, the traditional Chinese dietary pattern has gradually shifted toward the Western dietary pattern. This transition may contribute to the increased incidence of various diseases, including benign breast nodules (10, 11). Nevertheless, there is a lack of studies examining the association between dietary patterns and benign breast nodules in China.

This study aimed to investigate the association between dietary patterns and benign breast nodules in a community-based cross-sectional study in southeast China. Furthermore, we analyzed the association by subtypes of benign breast nodules and by menopausal status. This analysis may provide a foundation for precise interventions for benign breast nodules and primary prevention strategies for breast cancer.

## Materials and methods

### Study population

A total of 4,476 women aged 35–75 agreed to participate in the baseline survey of the Fuqing Cohort Study in the 23 villages of Gaoshan town in Southeast China from July 14, 2020 to June 31, 2021. The Fuqing cohort was designed to investigate the natural history and risk factors of non-communicable diseases among residents of the southeastern coastal region of China (12, 13). Women who underwent breast ultrasound examination were included, while those diagnosed with cancer or with a history of lumpectomy at enrollment were excluded, leaving 4,090 participants. All participants were interviewed face-to-face by trained staff using an electronic questionnaire developed by the Cohort Research Center of Fujian Medical University (https://cohort.fjmu.edu.cn/cobl). The questionnaire covered socio-demographic factors (age, education, occupation, family income, reproductive history, family history of cancers, and medical history) and lifestyle behaviors (cigarette smoking and alcohol consumption). The study protocol was approved by the Biomedical Ethics Review Committee of Fujian Medical University [approval numbers, (2017-07) and (2020-58)] and all participants provided written informed consent before enrollment.

### Questionnaire survey for dietary intake

Dietary intakes over the last year were assessed by a quantitative food frequency questionnaire (FFQ). The method used to evaluate the validity and reliability of the FFQ is described in a previous study in China (14). Generally, for food groups, the proportion of subjects classified into the same quartile by FFQs ranged from 70 to 87%, indicating moderate to strong agreement, and validity assessments showed moderate agreement (0.30–0.61) for most food groups between FFQ and 24-h dietary recall.

According to the food composition of our FFQ (Supplementary material), the total of 93 food items were grouped into 12 food categories (cereals, potatoes, vegetables, fruits, meat, aquatic products, eggs, dairy products, beans and soy products, nuts, snacks and desserts, and fried foods). Specific food items are listed in Supplementary Table 1. The frequency of intake (daily, weekly, monthly, yearly, or never) and the amount of each food item consumed by the study participants were collected using the common unit in the study area, liang (1 liang = 50 g). Daily intake (g/d) = each intake (g) × frequency of intake (days). The energy content (kcal/100 g) of each food item was obtained from the standardized version of the Chinese Food Composition Table (2018), 6th edition, and the total dietary energy intake was calculated (15). The residual method was applied to correct the influence of total energy intake and enhance the comparability of different individuals (16). Furthermore, women with energy intake < 600 kcal/day or >3,000 kcal/day (17), or those in the top 0.5% for the intakes of each food category were excluded as outliers. After these exclusions, 3,483 participants remained for our study (Supplementary Figure 1).

### Breast ultrasound measurement and definition of benign breast nodules

The breast ultrasound examination in the Fuqing cohort was conducted by qualified radiologists from the affiliated hospital of Fujian Medical University using the Aloka Prosound Alpha 7 ultrasound system. Following the guidelines for diagnosing breast disease using ultrasound in China (18), the identified benign breast nodules were categorized as cystic or solid nodules. Malignant breast nodules confirmed by pathological tests were classified as breast cancer and excluded from the study at enrollment. Additionally, we recorded the number of breast nodules and categorized the patients based on whether they had single or multiple nodules.

### Statistical analyses

Baseline characteristics of subjects with and without breast nodules were compared using Chi-square test (χ^2^). Principal component analysis (PCA) was conducted using the PROC FACTOR program in SAS to identify the major dietary components of the participants from 12 food groups. Extraction of dietary components was based on the eigenvalue screen plots (19). Factor scores for each dietary pattern were categorized into quartiles. Odds ratios (ORs) and 95% confidence intervals (CIs) were calculated to assess the association between quartiles of different dietary patterns and benign breast nodules using univariate and multivariate logistic regression models adjusted for age, body mass index, educational qualifications, family income, alcohol intake, number of births, age at menarche, menopausal status, age at first birth, ever use of oral contraceptive pill, ever use of hormone replacement therapy, and family history of breast cancer (shown in Table 1). Trend tests were applied to evaluate quartiles of each dietary pattern. Subgroup analyses by menopausal status were conducted for all benign breast nodules.

**Table 1:** Baseline characteristics of participants of the study^*^.

**Characteristics**	**Non-benign breast nodules (*N* = 3,038)**	**Benign breast nodules (*N* = 1,052)**	***χ^2^***	***P***
**Age at interview, y**			376.858	**< 0.001**
35~39	139 (0.05)	102 (0.10)		
40~49	440 (0.14)	364 (0.35)		
50~59	1,036 (0.34)	405 (0.38)		
60~69	1,115 (0.37)	153 (0.15)		
70–75	308 (0.10)	28 (0.03)		
**Body mass index, kg/m** ^2^			9.626	**0.022**
< 18.5	62 (0.02)	24 (0.02)		
18.5–23.9	1,444 (0.48)	529 (0.50)		
24.0–28.0	1,122 (0.37)	395 (0.38)		
>28.0	410 (0.13)	104 (0.10)		
**Educational status**			149.477	**< 0.001**
Illiterate	1,634 (0.54)	362 (0.34)		
Primary school	870 (0.29)	351 (0.33)		
Junior high/technical secondary school	355 (0.12)	240 (0.23)		
High school and above	122 (0.04)	76 (0.07)		
Unknown	57 (0.02)	23 (0.02)		
**Family income, 10,000 Yuan**			96.577	**< 0.001**
< 1	477 (0.16)	101 (0.10)		
1~	658 (0.22)	134 (0.13)		
3~	682 (0.22)	262 (0.25)		
6~	406 (0.13)	178 (0.17)		
≥10	567 (0.19)	298 (0.28)		
Unknown	248 (0.08)	79 (0.08)		
**Cigarette smoking**			4.531	0.104
No	2,957 (0.97)	1,015 (0.96)		
Yes	34 (0.01)	21 (0.02)		
Unknown	47 (0.02)	16 (0.02)		
**Alcohol drinking**			0.533	0.766
No	2,889 (0.95)	1,006 (0.96)		
Yes	103 (0.03)	31 (0.03)		
Unknown	46 (0.02)	15 (0.01)		
**Breastfeeding, month**			184.863	**< 0.001**
≤ 12	306 (0.10)	212 (0.20)		
~24	730 (0.24)	370 (0.35)		
~36	780 (0.26)	253 (0.24)		
>36	1,222 (0.40)	217 (0.21)		
**Number of abortions**			9.117	**0.010**
0	2,099 (0.69)	684 (0.65)		
1	592 (0.19)	213 (0.20)		
≥2	347 (0.11)	155 (0.15)		
**Age at first birth, y**			16.217	**0.001**
< 23	1,717 (0.57)	521 (0.50)		
23–27	1,162 (0.38)	466 (0.44)		
>27	93 (0.03)	42 (0.04)		
Unknown	66 (0.02)	23 (0.02)		
**Number of births**			87.169	**< 0.001**
≤ 1	170 (0.06)	105 (0.10)		
2	1,334 (0.44)	581 (0.55)		
≥3	1,474 (0.49)	345 (0.33)		
Unknown	60 (0.02)	21 (0.02)		
**Age at menarche, y**			86.088	**< 0.001**
< 13	55 (0.02)	36 (0.03)		
13–15	696 (0.23)	381 (0.36)		
>15	2,227 (0.73)	617 (0.59)		
Unknown	60 (0.02)	18 (0.02)		
**Menopausal status**			340.472	**< 0.001**
Premenopause	600 (0.20)	517 (0.49)		
Postmenopause	2,386 (0.79)	521 (0.50)		
Unknown	52 (0.02)	14 (0.01)		
**Age at menopause, y**			322.241	**< 0.001**
< 45	320 (0.11)	68 (0.06)		
45–50	1,212 (0.40)	246 (0.23)		
>50	854 (0.28)	207 (0.20)		
Unknown	652 (0.21)	531 (0.50)		
**Oophorectomy**			1.449	0.485
No	2,877 (0.95)	1,006 (0.96)		
Yes	109 (0.04)	32 (0.03)		
Unknown	52 (0.02)	14 (0.01)		
**Ever use of hormone replacement therapy**			22.795	**< 0.001**
No	2,804 (0.92)	930 (0.88)		
Yes	114 (0.04)	77 (0.07)		
Unknown	120 (0.04)	45 (0.04)		
**Ever use oral contraceptives**			2.680	0.262
No	2,949 (0.97)	1,019 (0.97)		
Yes	37 (0.01)	19 (0.02)		
Unknown	52 (0.02)	14 (0.01)		
**Family history of breast cancer**			6.623	**0.036**
No	2,938 (0.97)	1,011 (0.96)		
Yes	8 (0.00)	9 (0.01)		
Unknown	92 (0.03)	32 (0.03)		

Values are presented as *n*. ^*^Chi-square test for comparison of categorical variables. Statistically significant results are in bold.

All data analyses were performed using Stata15.1 and SAS 9.4, and all *P*-values were derived from two-sided tests with a significance level of α = 0.05.

## Results

### Baseline characteristics of the participants

Among all the recruited participants, 415 (10.15%) participants had cystic breast nodules, and 637 (15.57%) participants had solid breast nodules. Compared to participants without benign breast nodules, subjects with benign breast nodules were more likely to be between 40 and 60 years old, obese, with junior high school education, lower family income (< ¥100,000), breastfeeding for < 24 months, having more miscarriages, older at first birth, having fewer children, experiencing earlier menarche, later menopause, using hormone replacement therapy, and having a family history of breast cancer (Table 1).

### Dietary patterns

**Figure 1:**
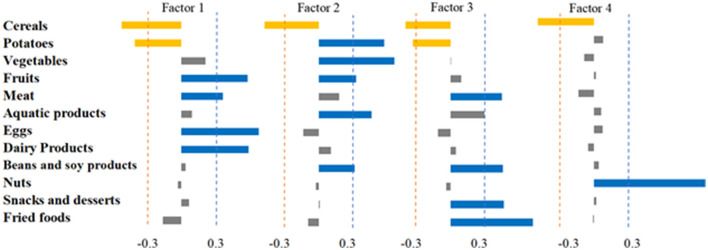
Dietary composition was analyzed using factor analysis to derive four dietary pattern factor loadings. The absolute value of the factor loadings >0.3 was used as the threshold value.

The PCA analysis identified four dietary patterns. Pattern 1 was “animal-based dietary pattern,” characterized by high consumption of eggs, dairy products, meat, and fruits. Dietary pattern 2 was the “plant-based dietary pattern,” with high intake of vegetables, potatoes, fruits, aquatic products, and beans and soy products. Dietary pattern 3 was characterized by high consumption of fried foods, snacks and desserts, beans and soy products, and meat, and was labeled “fried foods/dessert dietary pattern.” Dietary Pattern 4 had a notably high intake of nuts, which was named as the “nuts dietary pattern” (Figure 1).

### The associations between dietary patterns and benign breast nodules

Adjusted analyses showed a positive association between animal-based dietary patterns and benign breast nodules (*P* trend = 0.046), with OR = 1.28, 95% CI = 1.00–1.65 for the highest quartile group (Table 2). Further investigation into the associations by subtypes of benign breast nodules showed that animal-based dietary patterns were positively associated with breast cystic nodules, with ORs (95% CI) of 1.44 (1.01–2.06) and 1.61 (1.12–2.32) for the third and fourth quartile groups, respectively, with a significant trend (*P*-value for trend test = 0.007). Additionally, there was a positive association between fried foods/dessert dietary pattern and breast cystic nodules (*P*-value for trend test =0.012), with ORs (95% CI) for the third and highest quartiles of 1.45 (1.02–2.06), and 1.46 (1.01–2.09), respectively. No clear association was found between solid breast nodules and dietary patterns (Table 3).

**Table 2:** Logistic regression analysis of benign breast nodules prevalence risk with scores from various dietary patterns.

**Dietary patterns^#^**	**Non-Benign breast nodules**	**Case (n)**	**Univariate model**	Multivariate model^*^
	**(n)**		**OR (95%CI)**	**OR (95%CI)**
**Animal dietary pattern**				
Q1	709	161	1.00 (REF)	1.00 (REF)
Q2	672	199	1.12 (0.88–1.43)	1.11 (0.87–1.42)
Q3	625	246	1.21 (0.96–1.54)	1.19 (0.93–1.51)
Q4	555	316	**1.30 (1.02–1.65)**	**1.28 (1.00–1.65)**
*P trend*			**0.030**	**0.046**
**Plant dietary pattern**				
Q1	675	195	1.00 (REF)	1.00 (REF)
Q2	670	202	0.86 (0.68–1.09)	0.84 (0.66–1.06)
Q3	642	229	0.90 (0.71–1.14)	0.85 (0.67–1.08)
Q4	574	296	1.08 (0.86–1.36)	1.07 (0.84–1.35)
*P trend*			0.398	0.490
**Fried dessert dietary pattern**				
Q1	698	173	1.00 (REF)	1.00 (REF)
Q2	687	183	0.93 (0.73–1.18)	0.93 (0.73–1.19)
Q3	615	257	1.18 (0.94–1.49)	1.15 (0.90–1.45)
Q4	561	309	1.15 (0.90–1.46)	1.12 (0.88–1.44)
*P trend*			0.095	0.167
**Nut dietary pattern**				
Q1	676	195	1.00 (REF)	1.00 (REF)
Q2	666	204	0.89 (0.71–1.13)	0.87 (0.69–1.11)
Q3	624	248	1.04 (0.82–1.30)	1.00 (0.79–1.27)
Q4	595	275	1.01 (0.81–1.28)	0.98 (0.77–1.24)
*P trend*			0.594	0.828

^#^Grouped by quartiles of each modal score in the population; ^*^Adjusting factors: age, body mass index, educational qualifications, family income, alcohol intake, number of births, age at menarche, menopausal status, age at first birth, ever use of oral contraceptive pill use, ever use of hormone replacement therapy, family history of breast cancer. Participants without benign breast nodules as the reference. Statistically significant results are in bold.

**Table 3:** Logistic regression for the associations of cystic and solid breast nodules with different dietary patterns.

**Dietary patterns^#^**	**Cystic breast nodule**			**Solid breast nodule**		
	**Case**	**Univariate model**	**Multivariate model** ^*^	**Case**	**Univariate model**	**Multivariate model** ^*^
	**(n)**	**OR (95%CI)**	**OR (95%CI)**	**(n)**	**OR (95%CI)**	**OR (95%CI)**
**Animal dietary pattern**						
Q1	58	1.00 (REF)	1.00 (REF)	103	1.00 (REF)	1.00 (REF)
Q2	80	1.25 (0.87–1.79)	1.23 (0.85–1.78)	119	1.05 (0.78–1.40)	1.02 (0.76–1.37)
Q3	106	**1.47 (1.03–2.08)**	**1.44 (1.01–2.06)**	140	1.07 (0.80–1.42)	1.05 (0.78–1.41)
Q4	140	**1.61 (1.13–2.29)**	**1.61 (1.12–2.32)**	176	1.11 (0.83–1.49)	1.09 (0.80–1.48)
*P trend*		**0.006**	**0.007**		0.485	0.550
**Plant dietary pattern**						
Q1	67	1.00 (REF)	1.00 (REF)	128	1.00 (REF)	1.00 (REF)
Q2	92	1.15 (0.82–1.61)	1.10 (0.78–1.55)	110	**0.71 (0.54–0.95)**	**0.69 (0.52–0.93)**
Q3	98	1.13 (0.81–1.59)	1.04 (0.73–1.47)	131	0.78 (0.59–1.03)	**0.75 (0.56–1.00)**
Q4	127	1.33 (0.95–1.87)	1.31 (0.93–1.86)	169	0.92 (0.70–1.21)	0.92 (0.69–1.22)
*P trend*		0.113	0.153		0.813	0.779
**Fried dessert dietary pattern**						
Q1	61	1.00 (REF)	1.00 (REF)	112	1.00 (REF)	1.00 (REF)
Q2	74	1.06 (0.74–1.52)	1.04 (0.72–1.50)	109	0.86 (0.64–1.15)	0.86 (0.64–1.16)
Q3	112	**1.47 (1.04–2.07)**	**1.45 (1.02–2.06)**	145	1.01 (0.76–1.34)	0.98 (0.73–1.31)
Q4	137	**1.46 (1.03–2.07)**	**1.46 (1.01–2.09)**	172	0.97 (0.72–1.29)	0.94 (0.69–1.27)
*P trend*		**0.011**	**0.012**		0.907	0.905
**Nut dietary pattern**						
Q1	73	1.00 (REF)	1.00 (REF)	122	1.00 (REF)	1.00 (REF)
Q2	90	1.04 (0.74–1.45)	1.00 (0.71–1.41)	114	0.80 (0.60–1.07)	0.80 (0.59–1.06)
Q3	103	1.12 (0.80–1.56)	1.10 (0.78–1.54)	145	0.96 (0.73–1.27)	0.94 (0.71–1.25)
Q4	118	1.16 (0.83–1.61)	1.10 (0.78–1.55)	157	0.93 (0.70–1.22)	0.92 (0.69–1.22)
*P trend*		0.332	0.490		0.956	0.871

^#^Grouped by quartiles of each modal score in the population; ^*^Adjusting factors: age, body mass index, educational qualifications, family income, alcohol intake, number of births, age at menarche, menopausal status, age at first birth, ever use of oral contraceptive pill use, ever use of hormone replacement therapy, family history of breast cancer. Participants without benign breast nodules as the reference. Statistically significant results are in bold.

After stratifying the analysis by menopausal status, no statistically significant associations were observed between dietary patterns and benign breast nodules among premenopausal women. In postmenopausal women, those in the highest quartile group of the animal-based dietary pattern, were still associated with cystic breast nodules (OR = 1.90, 95% CI = 1.19–3.03, *P*-value for trend test = 0.011; Supplementary Table 2). A positive association between fried foods/dessert dietary pattern and cystic breast nodules was also present, with the *P*-value = 0.002 for the test of trend and an OR (95% CI) of 1.55 (1.01–2.39) for the third quartile group and 1.78 (1.12-2.82) for the highest quartile group, respectively.

According to the number of nodules detected using ultrasound, we further divided the patients into those with single and multiple nodules. However, results by the number of nodules showed no statistically significant associations between dietary patterns and breast nodules (Supplementary Table 3).

## Discussion

In the present study, we identified four dietary patterns among women in the southeastern coastal area of China, including animal, plant, fried food/dessert, and nuts dietary patterns. Among these dietary patterns, the animal-based dietary pattern and fried food/dessert pattern were significantly and positively associated with cystic breast nodules, and the association were also observed among postmenopausal women. None of the dietary patterns were associated with solid breast nodules.

Most of the existing studies focus on the association between dietary patterns and breast cancer. They show that a “healthy” or Mediterranean dietary pattern (characterized by high consumption of fruits, vegetables, whole grains, and soy products) has a protective effect against breast cancer. In contrast, an “unhealthy” or Western diet (marked by high consumption of meat, processed meats, saturated fats, refined grains, sweets, and desserts) is associated with an increased risk of breast cancer (20, 21). However, few studies have investigated the association with benign breast nodules, particularly cystic and solid breast nodules.

In our study, an animal-based dietary pattern was positively associated with benign breast nodules, consistent with previous findings (9). Our study further found that this dietary pattern was associated with an increased likelihood of breast cystic nodules, particularly in postmenopausal women. Previous studies have suggested that meat intake increases the risk of breast cancer due to carcinogens such as heterocyclic amines, N-nitroso compounds, and heme iron (22). Animal-based food (such as red meat and dairy products), are high in saturated fat and cholesterol, components that have been linked to an increased risk of breast cancer. Saturated fat promotes estrogen synthesis in the body, and high estrogen levels are an important factor in the development of breast cancer (23, 24). In addition, certain hormones and growth factors in animal foods may also act directly on the breast tissue and promote its abnormal proliferation (25). The underlying mechanism for the association with benign breast diseases might be similar to that for breast cancer. Previous case-control studies have also shown that iron in red meat increases plasma ferritin concentrations and further promotes cystic changes in the fibers of breast epithelial cells (26). Additionally, dairy products, eggs, and red meat are important sources of sex steroid hormones and insulin-like growth factor (27, 28), and their association with benign breast diseases is likely through a mechanism that affects these hormones (29, 30).

Some studies have suggested that a healthy dietary pattern based on fruits and vegetables is associated with a lower risk of benign breast nodules (9, 10). Fruits and vegetables are rich in antioxidants (e.g., vitamins C and E), fiber, and other bioactive components (31–33) that regulate the metabolism of hormones and carcinogens and inhibit the abnormal proliferation of breast cells. However, we did not find an inverse association between plant-based dietary pattern and benign breast nodules, probably because the vegetables were usually cooked with aquatic food together in the identified dietary pattern, which might attenuate the effect.

Fried foods and desserts are known to be high-energy and high-fat foods. Studies have shown that their consumption is associated with an increased risk of benign breast disease (10, 34, 35). Additionally, we found that a dietary pattern rich in fried foods and desserts was associated with breast cystic nodules, consistent with an increase in breast lesions (34). High-fat diets are correlated with elevated circulating estrogen levels (36). Moreover, high-temperature processing of protein-rich foods generates harmful substances, like heterocyclic amines and polycyclic aromatic hydrocarbons, both of which can elevate the likelihood of developing benign breast nodules (37, 38).

This study investigated the relationship between dietary patterns and benign breast nodule subtypes to offer personalized dietary recommendations for prevention or management. However, our study still has some limitations. First, it is a cross-sectional study that utilized the baseline survey of the Fuqing cohort study, which does not establish causality. The results of this study should be confirmed in a large prospective cohort. Second, the food frequency questionnaire assessed participants' past eating habits, which could introduce recall and information bias. To mitigate this bias, our participants underwent face-to-face interviews conducted by trained local staff, using visual aids depicting food in various portion sizes.

## Conclusions

This study identified four dietary patterns among women in the southeastern coastal area of China: animal-based, plant-based, fried foods/dessert, and nuts dietary patterns. The animal-based dietary pattern and fried food/dessert pattern showed a positive association with breast cystic nodules. Consuming an appropriate amount of meat and reducing the intake of fried foods/dessert may aid in preventing benign breast nodules and lowering the risk of breast cancer. Nevertheless, additional longitudinal studies are required in the future to validate our findings.

## Data Availability

The datasets presented in this article are not readily available because the local law of China. Requests to access the datasets should be directed to ywm@fjmu.edu.cn.
