# Supplementary material for: Dietary patterns associated with benign breast nodules by subtypes: a cross-sectional study in southeast China
Source: Front Nutr. 2025 Mar 25;12:1500853. doi: 10.3389/fnut.2025.1500853 (PMC11975567; doi:10.3389/fnut.2025.1500853)
Supplement: Supplementary file 1 [file Data_Sheet_1.docx]

**Dietary Patterns Associated with Benign Breast Nodules by Subtypes: A** **Cross-Sectional Study in Southeast China**

**Supplementary materials**

Supplementary Table 1. Food items in the dietary survey

Supplementary Table 2. Logistic regression for the associations of cystic and solid breast nodules with different dietary patterns, by menopausal status

Supplementary Table 3. Logistic regression analysis of different dietary pattern scores on the risk of single and multiple breast nodules

Supplementary Figure 1. Flow chart of inclusion of the study population.

Supplementary questionnaire : Food Frequency Questionnaire in Fuqing Cohort

Supplementary Table 1. Food items in the dietary survey

| **Food groups** | **Food names** |
| --- | --- |
| **Staple foods** | **Rice, porridge, rice noodles or vermicelli, pasta (noodles), steamed bread, meat buns, vegetable buns, corn, dumplings, wontons or meat swallows, kompyang, packed instant noodles, multi-grain porridge, buns (sweet potato filling), buns (peanut filling), oatmeal** |
| **Tubers** | **Potatoes** |
| **Eggs** | **Chicken eggs, duck eggs, quail eggs, goose eggs, salted eggs, preserved eggs** |
| **Meat** | **Pork, beef/lamb/rabbit, chicken/duck/goose, liver or chicken heart, other viscera, processed meats** |
| **Fish and fishery products** | **Freshwater fish, sea fish, squid or octopuses, shrimp, crabs, shellfish, jellyfish, sea tangle, laver, fish balls, dried fish/flatfish, dried shellfish(clam/oyster/scallop), Cured fish(salted fish)** |
| **Milk and dairy products** | **Fresh/boxed milk, yogurt, milk powder** |
| **Beans and soy products** | **Yellow beans, other dried beans, tofu, soybean milk, bean products, fried bean products, bean curd and bean paste** |
| **Vegetables** | **Legumes/fresh beans, solanaceae fruits, melon vegetables, onions and garlic, dark vegetables, light-colored vegetables, water vegetables, fungi, pickled vegetables** |
| **Fruits** | **Apples, pears, peaches, plums, winter/green jujube, grapes and raisins, strawberries, citrus/oranges/grapefruits, guavas, pineapples, longan, lychees, mangoes, bananas, papayas, loquats, pitaya, watermelons, cantaloupe, musk melon, ginseng fruit, kiwi fruit** |
| **Seeds and nuts** | **Tree nuts, dried fruits, peanuts, peanut soup/paste** |
| **Snacks and desserts** | **Western pastries, cookies, candy/compote/chocolate, ice cream** |
| **Fried foods** | **Fried pasta, fried potatoes (chips)** |

Supplementary Table 2. Logistic regression for the associations of cystic and solid breast nodules with different dietary patterns, by menopausal status

| **Menopausal states** | **Dietary patterns^#^** | **Non-Benign breast nodules** |  | **Cystic breast nodule** | | |  | **Solid breast nodule** | | |
| --- | --- | --- | --- | --- | --- | --- | --- | --- | --- | --- |
|  |  |  |  | **Case(n)** | **Univariate model** | **Multivariate model** |  | **Case(n)** | **Univariate model** | **Multivariate model** |
|  |  | **(n)** |  |  | **OR (95%CI)** | **OR (95%CI)** |  |  | **OR (95%CI)** | **OR (95%CI)** |
| **Premenopausal** |  |  |  |  |  |  |  |  |  |  |
|  | **Animal dietary pattern** | |  |  |  |  |  |  |  |  |
|  | Q1 | 47 |  | 17 | 1.00 (REF) | 1.00 (REF) |  | 24 | 1.00 (REF) | 1.00 (REF) |
|  | Q2 | 84 |  | 29 | 1.01 (0.50-2.05) | 0.90 (0.44-1.88) |  | 40 | 0.95 (0.51-1.76) | 0.91 (0.48-1.74) |
|  | Q3 | 133 |  | 54 | 1.19 (0.62-2.27) | 1.20 (0.61-2.34) |  | 68 | 1.02 (0.57-1.82) | 0.99 (0.54-1.80) |
|  | Q4 | 226 |  | 88 | 1.18 (0.63-2.20) | 1.10 (0.57-2.10) |  | 117 | 1.04 (0.60-1.81) | 1.04 (0.58-1.85) |
|  | *P trend* |  |  |  | 0.485 | 0.578 |  |  | 0.744 | 0.716 |
|  | **Plant dietary pattern** | |  |  |  |  |  |  |  |  |
|  | Q1 | 51 |  | 17 | 1.00 (REF) | 1.00 (REF) |  | 37 | 1.00 (REF) | 1.00 (REF) |
|  | Q2 | 104 |  | 45 | 1.35 (0.70-2.60) | 1.36 (0.69-2.69) |  | 48 | 0.64 (0.37-1.10) | 0.63 (0.35-1.11) |
|  | Q3 | 148 |  | 57 | 1.21 (0.64-2.27) | 1.21 (0.63-2.31) |  | 67 | 0.63 (0.37-1.05) | 0.62 (0.36-1.06) |
|  | Q4 | 187 |  | 69 | 1.20 (0.64-2.24) | 1.22 (0.64-2.34) |  | 97 | 0.72 (0.43-1.19) | 0.74 (0.44-1.25) |
|  | *P trend* |  |  |  | 0.928 | 0.872 |  |  | 0.480 | 0.596 |
|  | **Fried dessert dietary pattern** | |  |  |  |  |  |  |  |  |
|  | Q1 | 48 |  | 16 | 1.00 (REF) | 1.00 (REF) |  | 27 | 1.00 (REF) | 1.00 (REF) |
|  | Q2 | 73 |  | 33 | 1.40 (0.70-2.83) | 1.34 (0.65-2.78) |  | 31 | 0.76 (0.40-1.43) | 0.69 (0.36-1.31) |
|  | Q3 | 137 |  | 53 | 1.21 (0.63-2.32) | 1.18 (0.60-2.31) |  | 78 | 1.02 (0.59-1.77) | 1.00 (0.57-1.76) |
|  | Q4 | 232 |  | 86 | 1.20 (0.64-2.25) | 1.17 (0.61-2.26) |  | 113 | 0.88 (0.51-1.50) | 0.83 (0.47-1.45) |
|  | *P trend* |  |  |  | 0.948 | 0.934 |  |  | 0.896 | 0.840 |
|  | **Nut dietary pattern** | |  |  |  |  |  |  |  |  |
|  | Q1 | 53 |  | 19 | 1.00 (REF) | 1.00 (REF) |  | 36 | 1.00 (REF) | 1.00 (REF) |
|  | Q2 | 107 |  | 43 | 1.16 (0.61-2.18) | 1.15 (0.60-2.22) |  | 45 | 0.62 (0.36-1.08) | 0.60 (0.34-1.06) |
|  | Q3 | 145 |  | 52 | 1.05 (0.57-1.96) | 0.98 (0.52-1.87) |  | 74 | 0.76 (0.45-1.26) | 0.71 (0.42-1.21) |
|  | Q4 | 185 |  | 74 | 1.17 (0.65-2.12) | 1.14 (0.61-2.13) |  | 94 | 0.75 (0.46-1.24) | 0.74 (0.44-1.25) |
|  | *P trend* |  |  |  | 0.699 | 0.796 |  |  | 0.693 | 0.696 |
| **Postmenopausal** |  |  |  |  |  |  |  |  |  |  |
|  | **Animal dietary pattern** | |  |  |  |  |  |  |  |  |
|  | Q1 | 651 |  | 40 | 1.00 (REF) | 1.00 (REF) |  | 79 | 1.00 (REF) | 1.00 (REF) |
|  | Q2 | 583 |  | 51 | 1.37 (0.89-2.11) | 1.39 (0.90-2.16) |  | 77 | 1.04 (0.74-1.46) | 1.03 (0.73-1.45) |
|  | Q3 | 483 |  | 50 | 1.42 (0.92-2.21) | 1.42 (0.90-2.22) |  | 71 | 1.00 (0.70-1.42) | 1.00 (0.70-1.43) |
|  | Q4 | 325 |  | 51 | **1.91 (1.22-2.99)** | **1.90 (1.19-3.03)** |  | 57 | 1.07 (0.73-1.56) | 1.08 (0.73-1.60) |
|  | *P trend* |  |  |  | **0.006** | **0.011** |  |  | 0.802 | 0.772 |
|  | **Plant dietary pattern** | |  |  |  |  |  |  |  |  |
|  | Q1 | 614 |  | 49 | 1.00 (REF) | 1.00 (REF) |  | 90 | 1.00 (REF) | 1.00 (REF) |
|  | Q2 | 559 |  | 46 | 1.01 (0.66-1.54) | 0.97 (0.63-1.49) |  | 60 | 0.70 (0.49-0.99) | 0.71 (0.50-1.01) |
|  | Q3 | 487 |  | 40 | 0.97 (0.63-1.51) | 0.92 (0.59-1.45) |  | 64 | 0.83 (0.58-1.17) | 0.82 (0.57-1.17) |
|  | Q4 | 382 |  | 57 | **1.58 (1.05-2.39)** | **1.58 (1.03-2.41)** |  | 70 | 1.04 (0.73-1.47) | 1.07 (0.75-1.53) |
|  | *P trend* |  |  |  | **0.047** | 0.062 |  |  | 0.792 | 0.740 |
|  | **Fried dessert dietary pattern** | |  |  |  |  |  |  |  |  |
|  | Q1 | 640 |  | 43 | 1.00 (REF) | 1.00 (REF) |  | 85 | 1.00 (REF) | 1.00 (REF) |
|  | Q2 | 608 |  | 41 | 0.92 (0.59-1.44) | 0.92 (0.59-1.45) |  | 75 | 0.85 (0.61-1.19) | 0.87 (0.62-1.22) |
|  | Q3 | 471 |  | 58 | **1.54 (1.01-2.35)** | **1.55 (1.01-2.39)** |  | 67 | 0.89 (0.63-1.27) | 0.89 (0.62-1.27) |
|  | Q4 | 323 |  | 50 | **1.73 (1.11-2.70)** | **1.78 (1.12-2.82)** |  | 57 | 0.98 (0.67-1.43) | 1.01 (0.68-1.49) |
|  | *P trend* |  |  |  | **0.002** | **0.002** |  |  | 0.890 | 0.932 |
|  | **Nut dietary pattern** | |  |  |  |  |  |  |  |  |
|  | Q1 | 612 |  | 52 | 1.00 (REF) | 1.00 (REF) |  | 84 | 1.00 (REF) | 1.00 (REF) |
|  | Q2 | 553 |  | 47 | 0.96 (0.64-1.46) | 0.91 (0.60-1.39) |  | 69 | 0.89 (0.63-1.25) | 0.89 (0.63-1.27) |
|  | Q3 | 474 |  | 50 | 1.20 (0.79-1.81) | 1.14 (0.74-1.74) |  | 70 | 1.05 (0.74-1.48) | 1.05 (0.74-1.50) |
|  | Q4 | 403 |  | 43 | 1.09 (0.71-1.67) | 1.08 (0.69-1.68) |  | 61 | 0.94 (0.66-1.35) | 0.96 (0.67-1.39) |
|  | *P trend* |  |  |  | 0.486 | 0.529 |  |  | 0.984 | 0.948 |

Grouped by quartiles of each modal score in the population; ^*^ Adjusting factors: age, body mass index, educational qualifications, family income, alcohol intake, number of births, age at menarche, menopausal status, age at first birth, ever use of oral contraceptive pill use, ever use of hormone replacement therapy, family history of breast cancer. Participants without benign breast nodules as the reference.

Supplementary Table 3. Logistic regression analysis of different dietary pattern scores on the risk of single and multiple breast nodules

| **Dietary patterns** | **Non-nodule** | **Single of breast nodule** | | |  | **Multiple of breast nodule** | | |
| --- | --- | --- | --- | --- | --- | --- | --- | --- |
|  |  | **Case(n)** | **Univariate model** | **Multivariate model^*^** |  | **Case(n)** | **Univariate model** | **Multivariate model^*^** |
|  | **(n)** |  | **OR (95%CI)** | **OR (95%CI)** |  |  | **OR (95%CI)** | **OR (95%CI)** |
| **Animal**  **dietary pattern** |  |  |  |  |  |  |  |  |
| Q1 | 711 | 85 | 1.00 (REF) | 1.00 (REF) |  | 73 | 1.00 (REF) | 1.00 (REF) |
| Q2 | 676 | 90 | 1.00 (0.73-1.38) | 1.00 (0.72-1.38) |  | 102 | 1.20 (0.86-1.67) | 1.20 (0.86-1.68) |
| Q3 | 629 | 115 | 1.20 (0.88-1.64) | 1.21 (0.89-1.66) |  | 125 | 1.19 (0.86-1.65) | 1.18 (0.84-1.64) |
| Q4 | 559 | 119 | 1.12 (0.81-1.55) | 1.15 (0.82-1.61) |  | 192 | **1.42 (1.03-1.96)** | 1.37 (0.98-1.91) |
| *P trend* |  |  | 0.331 | 0.257 |  |  | **0.042** | 0.089 |
| **Plant**  **dietary pattern** |  |  |  |  |  |  |  |  |
| Q1 | 678 | 84 | 1.00 (REF) | 1.00 (REF) |  | 106 | 1.00 (REF) | 1.00 (REF) |
| Q2 | 673 | 109 | 1.15 (0.84-1.57) | 1.13 (0.83-1.54) |  | 89 | **0.65 (0.47-0.89)** | **0.63 (0.45-0.87)** |
| Q3 | 645 | 102 | 1.04 (0.76-1.42) | 1.02 (0.74-1.40) |  | 123 | 0.80 (0.59-1.08) | **0.73 (0.54-1.00)** |
| Q4 | 579 | 114 | 1.12 (0.82-1.55) | 1.16 (0.84-1.60) |  | 174 | 0.96 (0.72-1.29) | 0.93 (0.69-1.26) |
| *P trend* |  |  | 0.656 | 0.544 |  |  | 0.687 | 0.887 |
| **Fried dessert dietary pattern** |  |  |  |  |  |  |  |  |
| Q1 | 701 | 92 | 1.00 (REF) | 1.00 (REF) |  | 77 | 1.00 (REF) | 1.00 (REF) |
| Q2 | 692 | 80 | 0.80 (0.58-1.10) | 0.80 (0.58-1.10) |  | 95 | 1.04 (0.75-1.44) | 1.07 (0.76-1.49) |
| Q3 | 617 | 114 | 1.11 (0.82-1.51) | 1.11 (0.81-1.50) |  | 139 | 1.26 (0.92-1.73) | 1.23 (0.88-1.70) |
| Q4 | 565 | 123 | 1.05 (0.77-1.45) | 1.10 (0.79-1.52) |  | 181 | 1.22 (0.88-1.68) | 1.16 (0.83-1.62) |
| *P trend* |  |  | 0.349 | 0.256 |  |  | 0.146 | 0.308 |
| **Nut**  **dietary pattern** |  |  |  |  |  |  |  |  |
| Q1 | 678 | 95 | 1.00 (REF) | 1.00 (REF) |  | 97 | 1.00 (REF) | 1.00 (REF) |
| Q2 | 673 | 89 | 0.83 (0.61-1.14) | 0.81 (0.59-1.12) |  | 105 | 0.86 (0.63-1.18) | 0.86 (0.63-1.19) |
| Q3 | 625 | 121 | 1.14 (0.85-1.54) | 1.14 (0.84-1.54) |  | 125 | 0.92 (0.67-1.25) | 0.87 (0.63-1.2) |
| Q4 | 599 | 104 | 0.91 (0.66-1.24) | 0.91 (0.66-1.26) |  | 165 | 1.06 (0.79-1.42) | 1.01 (0.74-1.37) |
| *P trend* |  |  | 0.957 | 0.863 |  |  | 0.525 | 0.805 |

^#^ Grouped by quartiles of each modal score in the population; ^*^ Adjusting factors: age, body mass index, educational qualifications, family income, alcohol intake, number of births, age at menarche, menopausal status, age at first birth, ever use of oral contraceptive pill use, ever use of hormone replacement therapy, family history of breast cancer. Participants without benign breast nodules as the reference.

Supplementary Figure 1. Flow chart of inclusion of the study population.


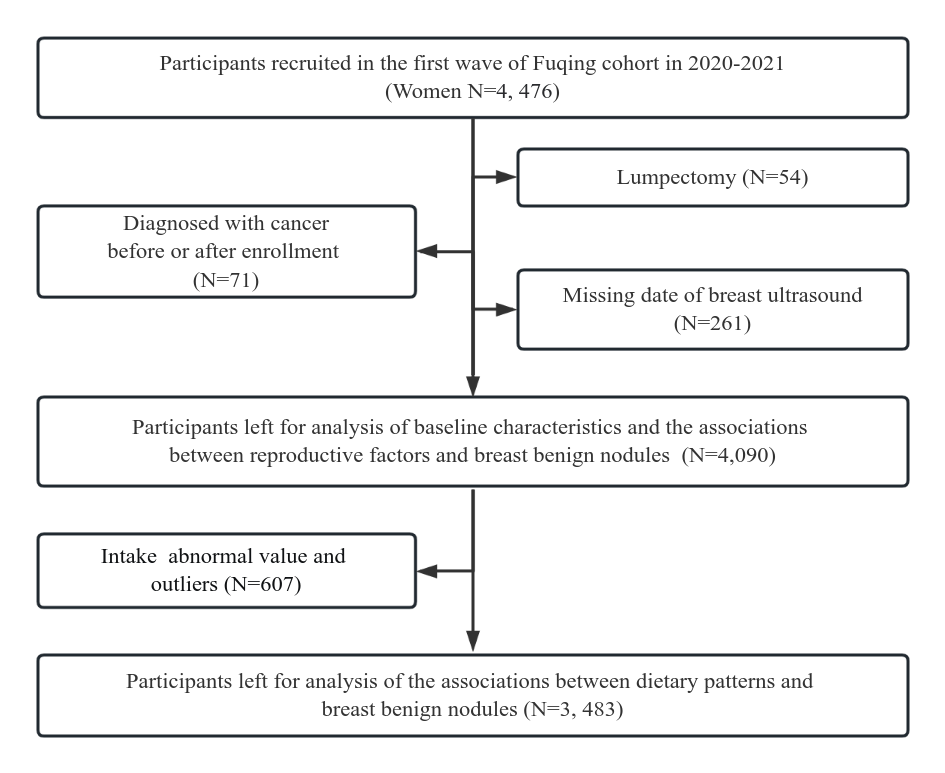


Supplementary questionnaire: Food Frequency Questionnaire in Fuqing Cohort

In the past year, how often have you consumed the following foods? (Frequency ≤6 times/ year is considered as not eating)

| Staple foods | Not eating | Frequency | Food portion sizes |
| --- | --- | --- | --- |
| Rice | □ | \|____\| ○times/day ○times/week ○times/month ○times/year | _____ liang |
| Porridge | □ | \|____\| ○times/day ○times/week ○times/month ○times/year | _____ liang |
| Rice noodles or vermicelli | □ | \|____\| ○times/day ○times/week ○times/month ○times/year | _____ liang |
| Pasta (noodles) | □ | \|____\| ○times/day ○times/week ○times/month ○times/year | _____ liang |
| Steamed bread | □ | \|____\| ○times/day ○times/week ○times/month ○times/year | _____ liang |
| Meat buns | □ | \|____\| ○times/day ○times/week ○times/month ○times/year | _____ liang |
| Vegetable buns | □ | \|____\| ○times/day ○times/week ○times/month ○times/year | _____ liang |
| Corn | □ | \|____\| ○times/day ○times/week ○times/month ○times/year | _____ liang |
| Dumplings | □ | \|____\| ○times/day ○times/week ○times/month ○times/year | _____ liang |
| Wontons or meat swallows | □ | \|____\| ○times/day ○times/week ○times/month ○times/year | _____ liang |
| Kompyang | □ | \|____\| ○times/day ○times/week ○times/month ○times/year | _____ liang |
| Packed instant noodles | □ | \|____\| ○times/day ○times/week ○times/month ○times/year | _____ liang |
| Multi-grain porridge | □ | \|____\| ○times/day ○times/week ○times/month ○times/year | _____ liang |
| Buns (sweet potato filling) | □ | \|____\| ○times/day ○times/week ○times/month ○times/year | _____ liang |
| Buns (peanut filling) | □ | \|____\| ○times/day ○times/week ○times/month ○times/year | _____ liang |
| Oatmeal | □ | \|____\| ○times/day ○times/week ○times/month ○times/year | _____ liang |
| Tubers | Not eating | Frequency | Food portion sizes |
| Tubers (sweet potato, potato, taro, Chinese yam) | □ | \|____\| ○times/day ○times/week ○times/month ○times/year | _____ liang |
| Eggs | Not eating | Frequency | Food portion sizes |
| Chicken eggs | □ | \|____\| ○times/day ○times/week ○times/month ○times/year | _____ liang |
| Duck eggs | □ | \|____\| ○times/day ○times/week ○times/month ○times/year | _____ liang |
| Quail eggs | □ | \|____\| ○times/day ○times/week ○times/month ○times/year | _____ liang |
| Goose eggs | □ | \|____\| ○times/day ○times/week ○times/month ○times/year | _____ liang |
| Salted eggs | □ | \|____\| ○times/day ○times/week ○times/month ○times/year | _____ liang |
| Preserved eggs | □ | \|____\| ○times/day ○times/week ○times/month ○times/year | _____ liang |
| Meat | Not eating | Frequency | Food portion sizes |
| Pork | □ | \|____\| ○times/day ○times/week ○times/month ○times/year | _____ liang |
| Beef/lamb/rabbit | □ | \|____\| ○times/day ○times/week ○times/month ○times/year | _____ liang |
| Chicken/duck/goose | □ | \|____\| ○times/day ○times/week ○times/month ○times/year | _____ liang |
| Liver or chicken heart | □ | \|____\| ○times/day ○times/week ○times/month ○times/year | _____ liang |
| Other viscera | □ | \|____\| ○times/day ○times/week ○times/month ○times/year | _____ liang |
| Processed meats | □ | \|____\| ○times/day ○times/week ○times/month ○times/year | _____ liang |
| Fish and fishery products | Not eating | Frequency | Food portion sizes |
| Freshwater fish | □ | \|____\| ○times/day ○times/week ○times/month ○times/year | _____ liang |
| Sea fish | □ | \|____\| ○times/day ○times/week ○times/month ○times/year | _____ liang |
| Squid or octopuses | □ | \|____\| ○times/day ○times/week ○times/month ○times/year | _____ liang |
| Shrimp | □ | \|____\| ○times/day ○times/week ○times/month ○times/year | _____ liang |
| Crabs | □ | \|____\| ○times/day ○times/week ○times/month ○times/year | _____ liang |
| Shellfish | □ | \|____\| ○times/day ○times/week ○times/month ○times/year | _____ liang |
| Jellyfish | □ | \|____\| ○times/day ○times/week ○times/month ○times/year | _____ liang |
| Sea tangle | □ | \|____\| ○times/day ○times/week ○times/month ○times/year | _____ liang |
| Laver | □ | \|____\| ○times/day ○times/week ○times/month ○times/year | _____ liang |
| Fish balls | □ | \|____\| ○times/day ○times/week ○times/month ○times/year | _____ liang |
| Dried fish/flatfish | □ | \|____\| ○times/day ○times/week ○times/month ○times/year | _____ liang |
| Dried shellfish(clam/oyster/scallop) | □ | \|____\| ○times/day ○times/week ○times/month ○times/year | _____ liang |
| Cured fish(salted fish) | □ | \|____\| ○times/day ○times/week ○times/month ○times/year | _____ liang |
| Milk and dairy products | Not eating | Frequency | Food portion sizes |
| Fresh/boxed milk | □ | \|____\| ○times/day ○times/week ○times/month ○times/year | _____ liang |
| Yogurt | □ | \|____\| ○times/day ○times/week ○times/month ○times/year | _____ liang |
| Milk powder | □ | \|____\| ○times/day ○times/week ○times/month ○times/year | _____ liang |
| Beans and soy products | Not eating | Frequency | Food portion sizes |
| Yellow beans | □ | \|____\| ○times/day ○times/week ○times/month ○times/year | _____ liang |
| Other dried beans | □ | \|____\| ○times/day ○times/week ○times/month ○times/year | _____ liang |
| Tofu | □ | \|____\| ○times/day ○times/week ○times/month ○times/year | _____ liang |
| Soybean milk | □ | \|____\| ○times/day ○times/week ○times/month ○times/year | _____ liang |
| Bean products | □ | \|____\| ○times/day ○times/week ○times/month ○times/year | _____ liang |
| Fried bean products | □ | \|____\| ○times/day ○times/week ○times/month ○times/year | _____ liang |
| Bean curd and bean paste | □ | \|____\| ○times/day ○times/week ○times/month ○times/year | _____ liang |
| Vegetables | Not eating | Frequency | Food portion sizes |
| Leguminous vegetables and sprout | □ | \|____\| ○times/day ○times/week ○times/month ○times/year  Consumed __ months per year | _____ liang |
| Solanaceous vegetables | □ | \|____\| ○times/day ○times/week ○times/month ○times/year | _____ liang |
| Melon vegetables | □ | \|____\| ○times/day ○times/week ○times/month ○times/year  Consumed __ months per year | _____ liang |
| Allium vegetables | □ | \|____\| ○times/day ○times/week ○times/month ○times/year | _____ liang |
| Dark vegetables | □ | \|____\| ○times/day ○times/week ○times/month ○times/year | _____ liang |
| Light-colored vegetables | □ | \|____\| ○times/day ○times/week ○times/month ○times/year | _____ liang |
| Aquatic vegetables | □ | \|____\| ○times/day ○times/week ○times/month ○times/year | _____ liang |
| Fungi | □ | \|____\| ○times/day ○times/week ○times/month ○times/year | _____ liang |
| Pickled vegetables | □ | \|____\| ○times/day ○times/week ○times/month ○times/year | _____ liang |
| Fruits | Not eating | Frequency | Food portion sizes |
| Apples | □ | \|____\| ○times/day ○times/week ○times/month ○times/year | _____ liang |
| Pears | □ | \|____\| ○times/day ○times/week ○times/month ○times/year | _____ liang |
| Peaches | □ | \|____\| ○times/day ○times/week ○times/month ○times/year  Consumed __ months per year | _____ liang |
| Plums | □ | \|____\| ○times/day ○times/week ○times/month ○times/year  Consumed __ months per year | _____ liang |
| Winter/green jujube | □ | \|____\| ○times/day ○times/week ○times/month ○times/year  Consumed __ months per year | _____ liang |
| Grapes and raisins | □ | \|____\| ○times/day ○times/week ○times/month ○times/year  Consumed __ months per year | _____ liang |
| Strawberries | □ | \|____\| ○times/day ○times/week ○times/month ○times/year  Consumed __ months per year | _____ liang |
| Citrus/oranges/grapefruits | □ | \|____\| ○times/day ○times/week ○times/month ○times/year | _____ liang |
| Guavas | □ | \|____\| ○times/day ○times/week ○times/month ○times/year | _____ liang |
| Pineapples | □ | \|____\| ○times/day ○times/week ○times/month ○times/year  Consumed __ months per year | _____ liang |
| Longan | □ | \|____\| ○times/day ○times/week ○times/month ○times/year  Consumed __ months per year | _____ liang |
| Lychees | □ | \|____\| ○times/day ○times/week ○times/month ○times/year  Consumed __ months per year | _____ liang |
| Mangoes | □ | \|____\| ○times/day ○times/week ○times/month ○times/year  Consumed __ months per year | _____ liang |
| Bananas | □ | \|____\| ○times/day ○times/week ○times/month ○times/year | _____ liang |
| Papayas | □ | \|____\| ○times/day ○times/week ○times/month ○times/year  Consumed __ months per year | _____ liang |
| Loquats | □ | \|____\| ○times/day ○times/week ○times/month ○times/year  Consumed __ months per year | _____ liang |
| Pitaya | □ | \|____\| ○times/day ○times/week ○times/month ○times/year  Consumed __ months per year | _____ liang |
| Watermelons | □ | \|____\| ○times/day ○times/week ○times/month ○times/year  Consumed __ months per year | _____ liang |
| Cantaloupe | □ | \|____\| ○times/day ○times/week ○times/month ○times/year  Consumed __ months per year | _____ liang |
| Musk melon | □ | \|____\| ○times/day ○times/week ○times/month ○times/year  Consumed __ months per year | _____ liang |
| Ginseng fruit | □ | \|____\| ○times/day ○times/week ○times/month ○times/year  Consumed __ months per year | _____ liang |
| Kiwi fruit | □ | \|____\| ○times/day ○times/week ○times/month ○times/year | _____ liang |
| Seeds and nuts | Not eating | Frequency | Food portion sizes |
| Tree nuts | □ | \|____\| ○times/day ○times/week ○times/month ○times/year | _____ liang |
| Dried fruit seeds | □ | \|____\| ○times/day ○times/week ○times/month ○times/year | _____ liang |
| Peanuts | □ | \|____\| ○times/day ○times/week ○times/month ○times/year | _____ liang |
| Peanut soup/paste | □ | \|____\| ○times/day ○times/week ○times/month ○times/year | _____ liang |
| Snacks and desserts | Not eating | Frequency | Food portion sizes |
| Western pastries | □ | \|____\| ○times/day ○times/week ○times/month ○times/year | _____ liang |
| Cookies | □ | \|____\| ○times/day ○times/week ○times/month ○times/year | _____ liang |
| Candy/compote/chocolate | □ | \|____\| ○times/day ○times/week ○times/month ○times/year | _____ liang |
| Ice cream | □ | \|____\| ○times/day ○times/week ○times/month ○times/year  Consumed __ months per year | _____ liang |
| Fried foods | Not eating | Frequency | Food portion sizes |
| Fried pasta | □ | \|____\| ○times/day ○times/week ○times/month ○times/year | _____ liang |
| Fried potatoes (chips) | □ | \|____\| ○times/day ○times/week ○times/month ○times/year | _____ liang |
